# Deletion of the Sequence Encoding the Tail Domain of the Bone Morphogenetic Protein type 2 Receptor Reveals a Bone Morphogenetic Protein 7-Specific Gain of Function

**DOI:** 10.1371/journal.pone.0076947

**Published:** 2013-10-08

**Authors:** Patricio A. Leyton, Hideyuki Beppu, Alexandra Pappas, Trejeeve M. Martyn, Matthias Derwall, David M. Baron, Rita Galdos, Donald B. Bloch, Kenneth D. Bloch

**Affiliations:** 1 Anesthesia Center for Critical Care Research, Department of Anesthesia, Critical Care and Pain Medicine, Massachusetts General Hospital and Harvard Medical School, Boston, Massachusetts, United States of America; 2 Department of Clinical Laboratory and Molecular Pathology, Graduate School of Medicine and Pharmaceutical Science, University of Toyama, Toyama, Toyama Prefecture, Japan; 3 Department of Anesthesiology, Uniklinik Aachen, RWTH Aachen University, Aachen, North Rhine-Westphalia, Germany; 4 Department of Anesthesia, General Intensive Care, and Pain Management, Medical University of Vienna, Vienna, Austria; 5 Center for Immunology and Inflammatory Diseases, Department of Medicine, Massachusetts General Hospital and Harvard Medical School, Boston, Massachusetts, United States of America; 6 Cardiovascular Research Center, Cardiology Division of the Department of Medicine, Massachusetts General Hospital and Harvard Medical School, Boston, Massachusetts, United States of America; University of Illinois College of Medicine, United States of America

## Abstract

The bone morphogenetic protein (BMP) type II receptor (BMPR2) has a long cytoplasmic tail domain whose function is incompletely elucidated. Mutations in the tail domain of BMPR2 are found in familial cases of pulmonary arterial hypertension. To investigate the role of the tail domain of BMPR2 in BMP signaling, we generated a mouse carrying a *Bmpr2* allele encoding a non-sense mediated decay-resistant mutant receptor lacking the tail domain of Bmpr2. We found that homozygous mutant mice died during gastrulation, whereas heterozygous mice grew normally without developing pulmonary arterial hypertension. Using pulmonary artery smooth muscle cells (PaSMC) from heterozygous mice, we determined that the mutant receptor was expressed and retained its ability to transduce BMP signaling. Heterozygous PaSMCs exhibited a BMP7‑specific gain of function, which was transduced via the mutant receptor. Using siRNA knockdown and cells from conditional knockout mice to selectively deplete BMP receptors, we observed that the tail domain of Bmpr2 inhibits Alk2‑mediated BMP7 signaling. These findings suggest that the tail domain of Bmpr2 is essential for normal embryogenesis and inhibits Alk2‑mediated BMP7 signaling in PaSMCs.

## Introduction

Bone morphogenetic proteins (BMPs) were initially identified as signaling factors involved in the formation of bone and cartilage. BMPs are now known to participate in a broad spectrum of biological activities during embryogenesis and organogenesis, as well as in the homeostasis of mature organs [1,2]. BMPs are members of the transforming growth factor beta family. BMPs bind to heterotetrameric receptor complexes formed by BMP type 2 and BMP type 1 serine–threonine kinases. Upon assembly of the BMP receptor complex by a BMP ligand, the constitutively active type 2 receptor phosphorylates the type 1 receptor, which in turn activates cytoplasmic BMP-responsive Smad signaling molecules—Smads 1, 5, and 8. Phosphorylated BMP-responsive Smads interact with Smad4 and translocate into the nucleus, where they modulate the transcription of BMP-responsive genes, such as *Id1* and *Smad6* [2-4].

BMP receptors include four type 1 (Alk1, Alk2, Alk3 and Alk6) and three type 2 kinases (Bmpr2, Acvr2a and Acvr2b) [2]. The expression of these receptors differs depending on the cell type or tissue. For example, mouse pulmonary artery smooth muscle cells (PaSMCs) express Bmpr2 and Acvr2a with lower amounts of Acvr2b; Alk2 and Alk3 are the predominant BMP type 1 receptors expressed in PaSMCs [5]. All BMP receptors have a similar structure including an extracellular ligand-binding domain, a transmembrane domain, and a cytoplasmic serine–threonine kinase domain. Unlike other BMP receptors, the predominantly expressed form of Bmpr2 (Bmpr2‑WT) contains a long cytoplasmic tail domain (Bmpr2‑TD) encoded by *Bmpr2* exons 12 and 13 [6,7]. In a small fraction of *Bmpr2* transcripts, exon 12 is alternatively spliced, resulting in a short-form variant of the receptor [7]. Although the Bmpr2‑TD has been reported to interact with several proteins that can modulate BMP signaling [8,9], the functional role of the Bmpr2‑TD remains to be fully defined.

*BMPR2* is implicated in the development of pulmonary arterial hypertension (PAH) [10,11]. PAH is a disease of the pulmonary circulation characterized by neointimal formation, obstruction of vessels, plexiform lesions, and pruning of the small pulmonary arteries [12]. Heterozygous *BMPR2* mutations have been reported in approximately 75% of patients with hereditary PAH and in 25% of idiopathic cases [13]. Seventy percent of *BMPR2* mutations introduce a premature termination codon [14]. *BMPR2* transcripts containing premature termination codons are subject to nonsense-mediated decay (NMD), an RNA surveillance mechanism that degrades aberrant mRNAs [15,16]. *BMPR2* transcripts that undergo NMD lead to functional haploinsufficiency. However, some types of mutant transcripts can escape NMD, and the translated mutant receptor may exhibit a more deleterious phenotype by acting, for example, in a dominant-negative manner. It has been reported that PAH has an earlier onset and a worse prognosis in patients that carry NMD-resistant *BMPR2* mutations than in patients who carry NMD-sensitive *BMPR2* mutations [17].

In previous studies, we examined the impact of *Bmpr2* haploinsufficiency on BMP signaling in PaSMCs isolated from genetically modified mice. We observed that PaSMCs isolated from heterozygous mice carrying a *Bmpr2* mutant allele lacking exons 4 and 5 (*Bmpr2* ^*+/-*^) were less responsive to BMP4 and BMP7 than were PaSMCs isolated from WT mice [5,18]. We also investigated the impact of complete loss of Bmpr2 using PaSMCs from mice harboring mutant *Bmpr2* alleles in which exon 4 and 5 were flanked with loxP sequences (*Bmpr2* ^*flox/flox*^) [5]. When both *Bmpr2* alleles were disrupted (*Bmpr2* ^*del/del*^) in PaSMCs, BMP4 signaling was diminished, whereas BMP7 signaling was unexpectedly increased. We found that Acvr2a, but not Acvr2b, compensated for the absence of Bmpr2.

To investigate the role of the tail domain of Bmpr2 and model the impact of an NMD-resistant *Bmpr2* mutation, we generated mice that carry a mutant *Bmpr2* allele (*Bmpr2* ^*Δtd*^), which encodes a receptor lacking the tail domain of Bmpr2 (Bmpr2‑ΔTD). We isolated PaSMCs from mice carrying the *Bmpr2* ^*Δtd*^ allele to characterize the role of Bmpr2-TD in BMP signaling. 

## Results

### Generation and phenotype of mutant mice harboring the Bmpr2^Δtd^ allele

The mutant *Bmpr2* ^*Δtd*^ allele was generated by inserting a cassette encoding the enhanced green fluorescent protein (Egfp) and a stop codon in frame after exon 11. The strategy for generating mice carrying the mutant *Bmpr2* ^*Δtd*^ allele is described in Methods and Figure S1. We observed that *Bmpr2* ^*Δtd/Δtd*^ mice died early in embryogenesis (embryonic day (E) 7.5 to 8.5), revealing a previously unknown role for the Bmpr2‑TD in embryogenesis. In contrast, *Bmpr2* ^*Δtd/+*^ mice grow normally and have a lifespan similar to that of their WT littermates. Right ventricular systolic pressure and mean arterial pressure did not differ between *Bmpr2* ^*Δtd/+*^ and WT mice at 6 to 8 months of age (Figure S2). These findings suggest that *Bmpr2* ^*Δtd/+*^ mice do not spontaneously develop PAH.

### Expression of Bmpr2‑ΔTD in PaSMCs

**Figure 1:**
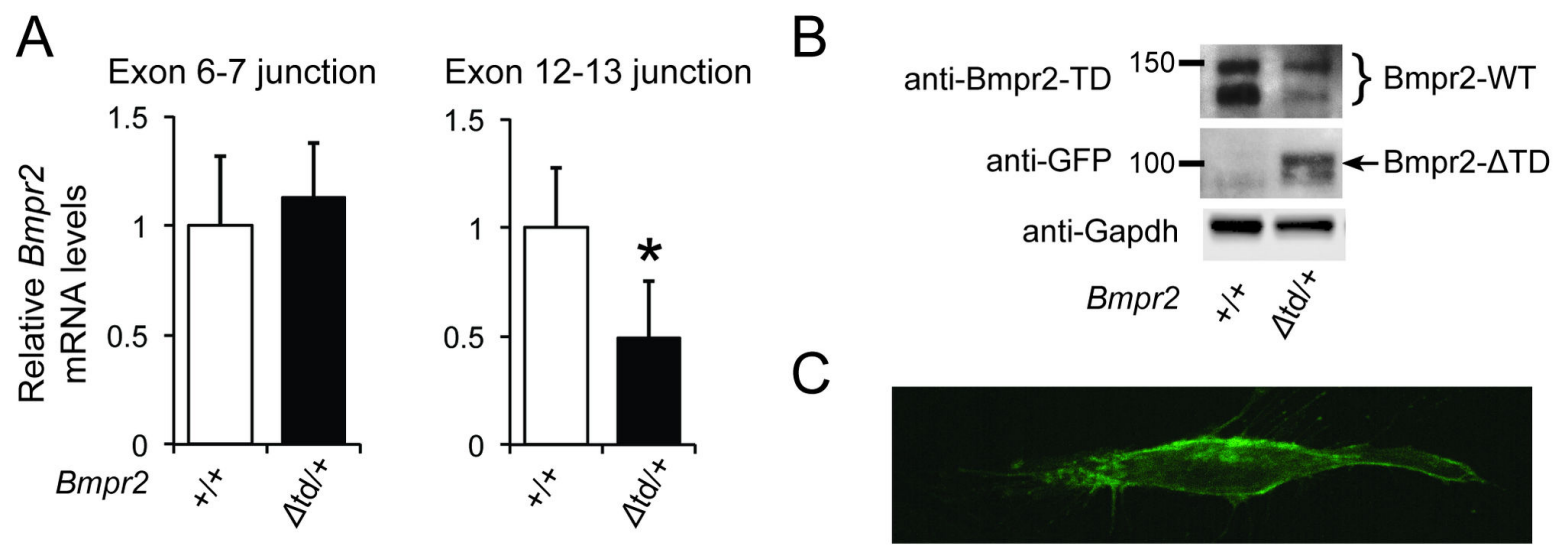
Bmpr2 expression in PaSMCs obtained from WT or *Bmpr2* ^*Δtd/+*^ mice. (A) Levels of *Bmpr2* mRNA were measured in WT (Bmpr2^+/+^) or *Bmpr2* ^*Δtd/+*^ PaSMCs by qPCR using hydrolysis probes for *Bmpr2* exon junctions 6–7 and 12–13. *Bmpr2* mRNA levels were normalized to *Gapdh* and expressed as the fold-change relative to *Bmpr2* ^*+/+*^ PaSMCs. *P < 0.01 compared to *Bmpr2* ^*+/+*^ PaSMCs. (B) Immunoblots prepared from lysates of *Bmpr2* ^*+/+*^ and *Bmpr2* ^*Δtd/+*^ PaSMCs were incubated with an antibody directed against the tail domain of Bmpr2 to detect Bmpr2‑WT or with an anti-GFP antibody to detect Bmpr2‑ΔTD. Immunoblots were subsequently incubated with an antibody directed against Gapdh as a control for protein loading. (C) Confocal microscopy image of a PaSMC transiently transfected with a plasmid directing expression of *Bmpr2* ^*Δtd*^ and reacted with an anti-GFP antibody showing localization of Bmpr2‑ΔTD at the cell membrane.

To begin to understand the impact of the *Bmpr2* ^*Δtd*^ allele on BMP signaling, we sought to determine whether the mutant *Bmpr2* gene is expressed. *Bmpr2* ^*+*^ and *Bmpr2* ^*Δtd*^ mRNA and protein levels were measured in PaSMCs using quantitative real-time PCR (qPCR) and immunoblot techniques, respectively. Total *Bmpr2* mRNA levels did not differ between WT and *Bmpr2* ^*Δtd/+*^ PaSMCs, when determined using oligonucleotides spanning the *Bmpr2* exon 6–7 junction (Figure 1A). *Bmpr2* ^*+*^ mRNA levels in *Bmpr2* ^*Δtd/+*^ PaSMCs were half of those observed in WT cells when measured using oligonucleotides spanning the exon 12–13 junction. Immunoblot analysis showed that levels of Bmpr2‑WT were less in *Bmpr2* ^*Δtd/+*^ PaSMCs than in WT PaSMCs. The protein expressed by the mutant allele, Bmpr2‑ΔTD, in *Bmpr2* ^*Δtd/+*^ cells was smaller (~100 kDa) than Bmpr2‑WT (Figure 1B). These results show that the *Bmpr2* ^*Δtd*^ allele is transcribed, *Bmpr2* ^*Δtd*^ transcripts are resistant to NMD, and Bmpr2‑ΔTD protein is expressed in PaSMCs.

To determine whether Bmpr2‑ΔTD can localize to the cell membrane, PaSMCs were transfected with a plasmid directing the expression of *Bmpr2* ^*Δtd*^, following by immunostaining with an antibody directed against GFP. Confocal microscopy revealed that the mutant receptor localized to the cell membrane (Figure 1C). This finding suggests that the Bmpr2‑TD is not required for intracellular trafficking of Bmpr2‑WT to the cell membrane.

### Bmpr2^Δtd/+^ PaSMCs exhibit a BMP ligand-specific gain of function

**Figure 2:**
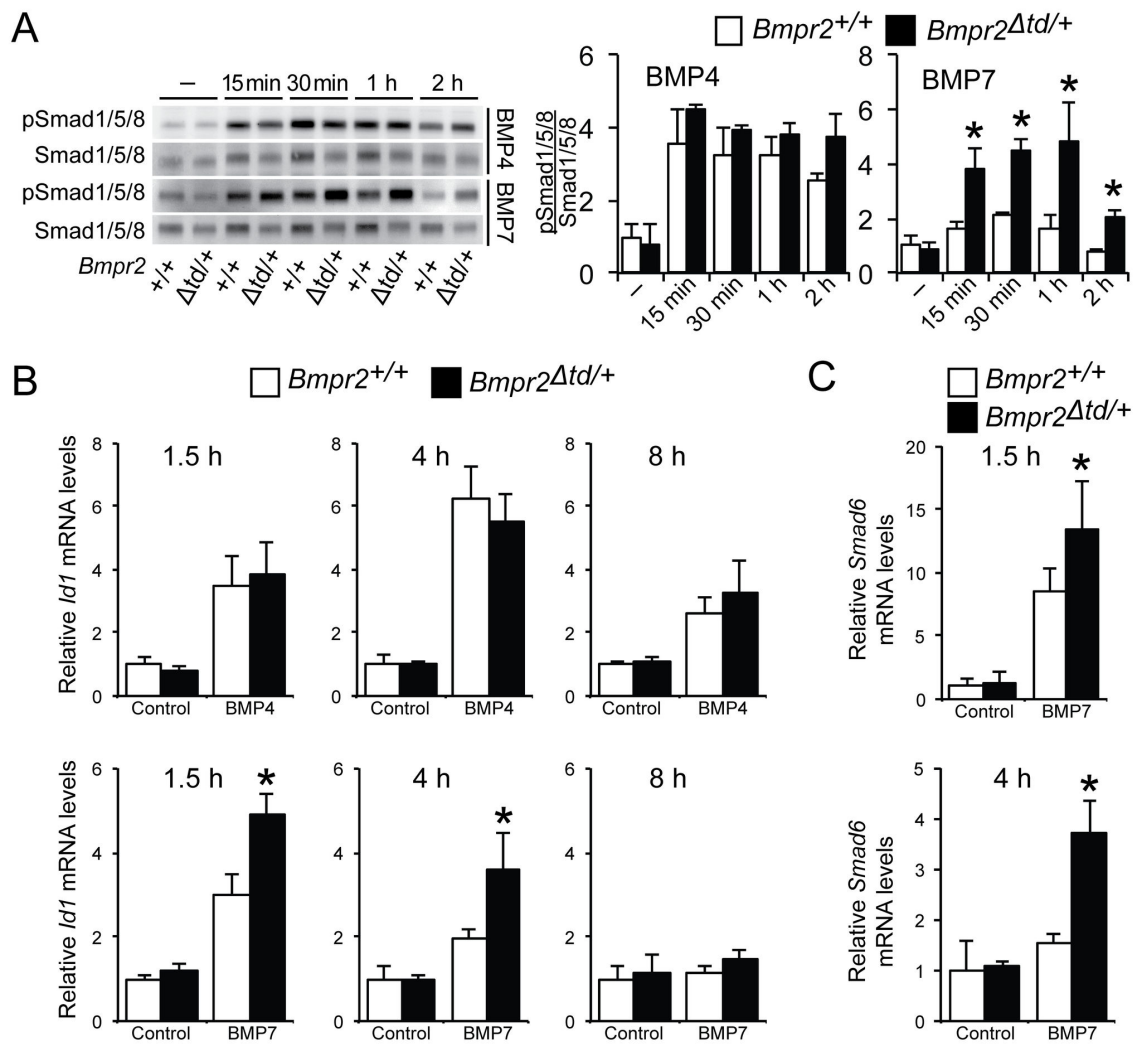
BMP7 signaling is enhanced in *Bmpr2* ^*Δtd/+*^ PaSMCs. (A) Immunoblots of lysates of WT (Bmpr2^+/+^) or *Bmpr2* ^*Δtd/+*^ PaSMCs treated with BMP4 or BMP7 (10 ng/ml) for various times were reacted with antibodies directed against phosphorylated and total Smad1/5/8. Quantification of the ratio of phosphorylated Smad1/5/8 to total Smad1/5/8 (analysis of 3 independent experiments) demonstrated that BMP4 signaling is similar in *Bmpr2* ^*+/+*^ or *Bmpr2* ^*Δtd/+*^ PaSMCs, whereas BMP7 signaling is greater in *Bmpr2* ^*Δtd/+*^ PaSMCs. *P<0.05 compared to *Bmpr2* ^*+/+*^ PaSMC group treated with BMP7. *Id1* (B) and *Smad6* (C) mRNA levels were measured by qPCR in *Bmpr2* ^*+/+*^ or *Bmpr2* ^*Δtd/+*^ PaSMCs treated with BMP4 or BMP7 (10 ng/ml) for various times. *Id1* and *Smad6* gene expression was normalized to *Gapdh* and expressed as fold-change relative to control *Bmpr2* ^*+/+*^ PaSMC group. *P < 0.01 compared to *Bmpr2* ^*+/+*^ PaSMC group treated with BMP7.

The observation that Bmpr2‑ΔTD localizes to the cell membrane suggested that the mutant receptor could participate in BMP signaling. To investigate the impact of the Bmpr2‑TD on BMP signaling, we compared PaSMCs from WT and *Bmpr2* ^*Δtd/+*^ mice. Incubation with BMP4 induced the phosphorylation of Smad1/5/8 and expression of the *Id1* gene similarly in WT and *Bmpr2* ^*Δtd/+*^ PaSMCs (Figure 2A and B). In both WT and *Bmpr2* ^*Δtd/+*^ PaSMC, BMP4 induction of *Id1* gene expression peaked at 4 hours (Figure 2A) and persisted for up to 24 hours (Figure S3). In contrast, incubation with BMP7 for 1.5 and 4 hours led to a greater induction of Smad1/5/8 phosphorylation and *Id1* and *Smad6* gene expression in *Bmpr2* ^*Δtd/+*^ PaSMCs than in WT cells (Figure 2B and C). After 8 hours of exposure to BMP7, levels of *Id1* mRNA returned to baseline (Figure 2B). These findings suggest that loss of one copy of the Bmpr2‑TD leads to a BMP ligand-specific gain of function.

### Bmpr2‑ΔTD contributes to the increased responsiveness of Bmpr2^Δtd/+^ PaSMCs to BMP7

**Figure 3:**
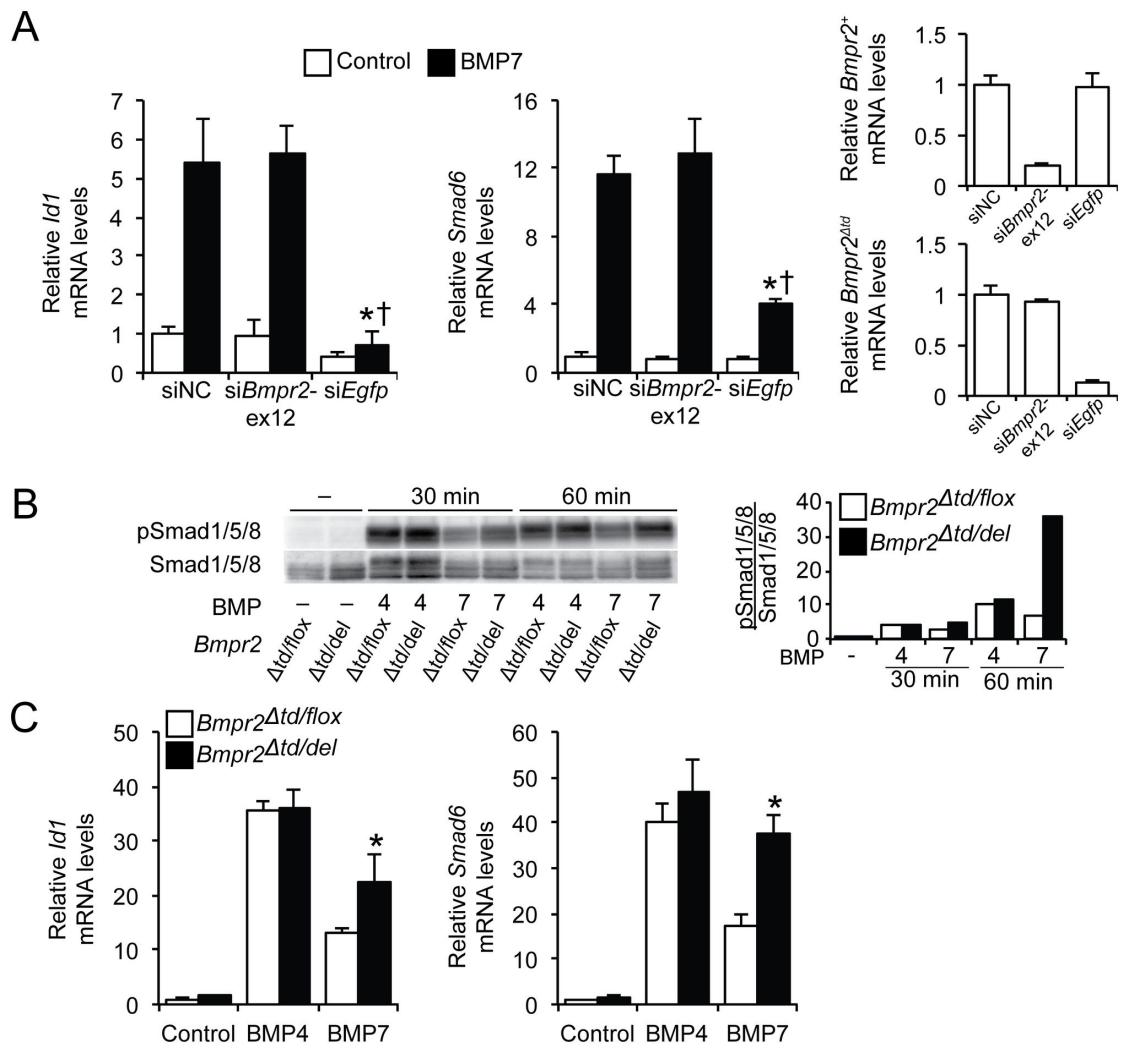
Bmpr2‑ΔTD contributes to BMP7 signaling in *Bmpr2* ^*Δtd/+*^ PaSMCs. (A) *Bmpr2* ^*Δtd/+*^ PaSMCs were transfected with negative control siRNA (siNC), si*Bmpr2*‑ex12, or si*Egfp* (30 nM). After 48 h, the ability of BMP7 (10 ng/ml for 1.5 h) to induce *Id1* and *Smad6* mRNA expression was measured by qPCR, normalized to *Gapdh* and expressed as fold-change relative to *Bmpr2* ^*Δtd/+*^ PaSMCs transfected with siNC. *P < 0.01 compared to siNC group treated with BMP7, ^†^ P<0.01 compared to si*Bmpr2*‑ex12 group treated with BMP7. Efficiency of silencing *Bmpr2* ^*+*^ (si*Bmpr2*‑ex12) and *Bmpr2* ^*Δtd*^ (si*Egfp*) transcripts was measured by qPCR. (B) *Bmpr2* ^*Δtd/flox*^ and *Bmpr2* ^*Δtd/del*^ PaSMCs were treated with BMP4 or BMP7 (10 ng/ml) for 30 and 60 minutes, upon which the activation of Smad1/5/8 was evaluated by immunoblotting. Quantification of the Smad1/5/8 activation is plotted as the ratio of pSmad1/5/8 to total Smad1/5/8. (C) The ability of BMP4 or BMP7 to induce *Id1* and *Smad6* gene expression in *Bmpr2* ^*Δtd/flox*^ and *Bmpr2* ^*Δtd/del*^ PaSMCs was measured by qPCR, normalized to *Gapdh* and expressed as fold-change relative to untreated *Bmpr2* ^*Δtd/flox*^ PaSMCs. *P < 0.01 compared to *Bmpr2* ^*Δtd/flox*^ PaSMC group treated with BMP7.

To investigate whether the enhanced responsiveness of *Bmpr2* ^*Δtd/+*^ PaSMCs to BMP7 depends on the presence of Bmpr2‑ΔTD, *Bmpr2* ^*Δtd/+*^ PaSMCs were treated with small interfering RNAs (siRNAs) to silence *Bmpr2* ^*+*^ mRNA (targeting *Bmpr2* exon 12; si*Bmpr2‑ex12*) or *Bmpr2* ^*Δtd*^ mRNA (targeting *Egfp*; si*Egfp*). The ability of BMP7 to induce *Id1* and *Smad6* gene expression was retained in *Bmpr2* ^*Δtd/+*^ PaSMCs treated with si*Bmpr2‑ex12*, but decreased in cells treated si*Egfp* (Figure 3A). These data suggest that the enhanced BMP7 signaling seen in *Bmpr2* ^*Δtd/+*^ PaSMCs requires Bmpr2‑ΔTD.

To confirm that the increased BMP7 signaling seen in *Bmpr2* ^*Δtd/+*^ cells does not require expression of the wild-type allele, we infected PaSMCs from *Bmpr2* ^*Δtd/flox*^ mice with an adenovirus specifying Cre recombinase (Ad-Cre) to delete the *Bmpr2* ^*flox*^ allele (*Bmpr2* ^*Δtd/del*^ PaSMCs). *Bmpr2* ^*Δtd/flox*^ cells infected with an adenovirus specifying red fluorescent protein (Ad-RFP) were used as control. *Bmpr2* ^*Δtd/del*^ PaSMCs did not express detectable Bmpr2‑WT protein (Figure S4). Incubation with BMP4 led to a similar induction of Smad1/5/8 phosphorylation (Figure 3B) and *Id1* and *Smad6* gene expression (Figure 3C) in *Bmpr2* ^*Δtd/flox*^ and *Bmpr2* ^*Δtd/del*^ PaSMCs. In contrast, incubation with BMP7 led to a greater increase in the phosphorylation of Smad1/5/8 and in *Id1* and *Smad6* gene expression in *Bmpr2* ^*Δtd/del*^ than in *Bmpr2* ^*Δtd/flox*^ PaSMCs. These results provide additional support for the concept that the presence of Bmpr2‑WT is not required for *Bmpr2* ^*Δtd/flox*^ PaSMCs to signal in response to BMP7. Moreover, the presence of the *Bmpr2* ^*+*^ allele appears to inhibit signaling via Bmpr2‑ΔTD.

**Figure 4:**
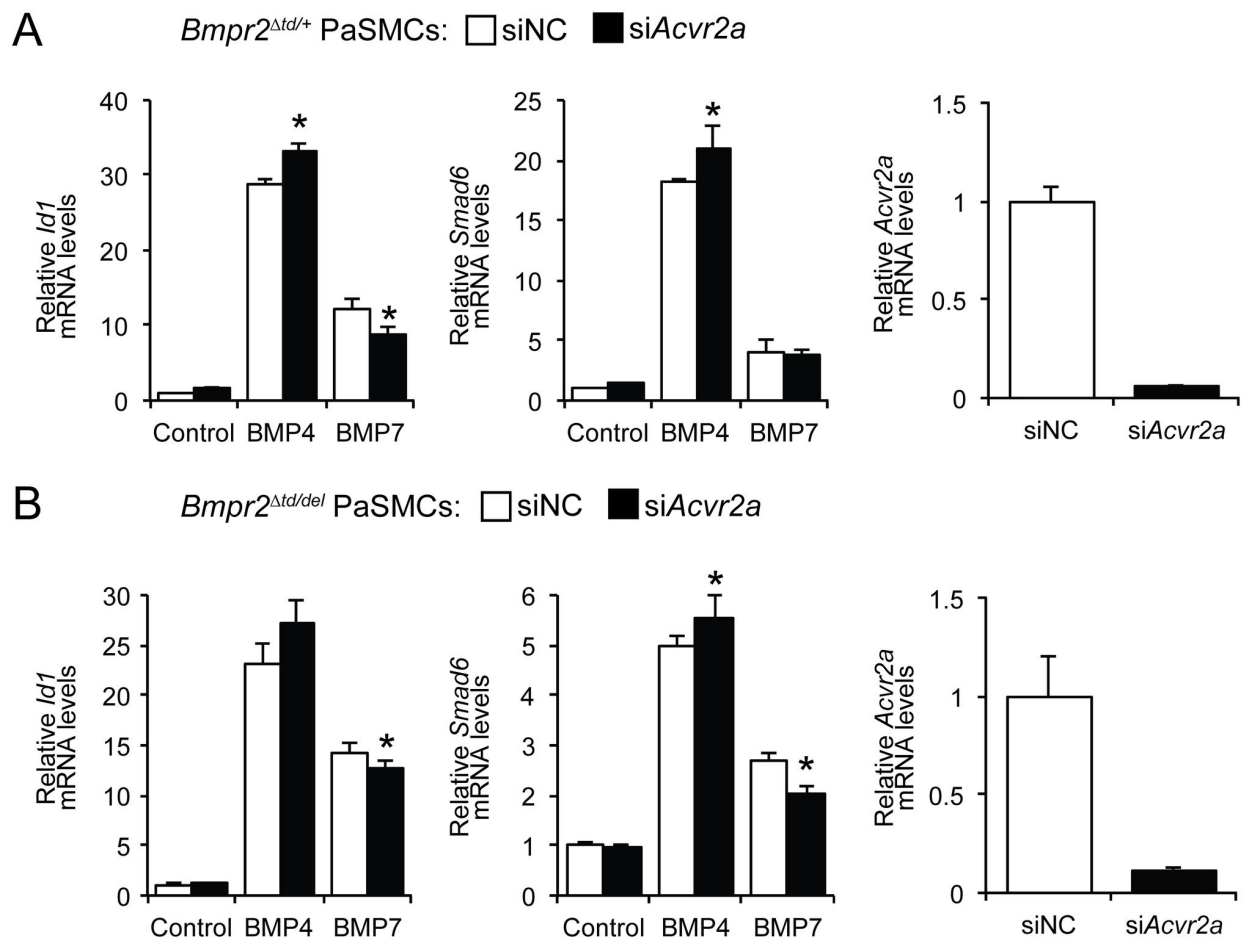
BMP7 signaling in *Bmpr2* ^*Δtd/+*^ and *Bmpr2* ^*Δtd/del*^ PaSMCs does not depend on the presence of Acvr2a. (A) *Bmpr2* ^*Δtd/+*^ PaSMCs were treated with a siRNA specific for *Acvr2a* transcripts. The ability of BMP4 or BMP7 (10 ng/ml for 1.5 h) to induce *Id1* and *Smad6* gene expression was measured by qPCR, normalized to *Gapdh* and expressed as fold-change relative to *Bmpr2* ^*Δtd/+*^ PaSMCs treated with siNC. *P < 0.01 compared to siNC within BMP treatment. Silencing efficiency was quantified by measuring *Acvr2a* mRNA levels. (B) *Bmpr2* ^*Δtd/del*^ PaSMCs were treated with si*Acvr2a*. The ability of BMP4 or BMP7 (10 ng/ml for 1.5 h) to induce *Id1* and *Smad6* gene expression was measured by qPCR, normalized to *Gapdh* and expressed as fold-change relative to *Bmpr2* ^*Δtd/del*^ PaSMCs treated with siNC. *P < 0.01 compared to siNC within BMP treatment. *Acvr2a* silencing efficiency was measured by qPCR.

Since Acvr2a, but not Acvr2b, can compensate for the absence of Bmpr2 in *Bmpr2* ^*del/del*^ PaSMCs [5], we considered the possibility that Acvr2a was responsible for BMP signaling in *Bmpr2* ^*Δtd/+*^ or *Bmpr2* ^*Δtd/del*^ PaSMCs. Silencing *Acvr2a* mRNA modestly increased the ability of BMP4 to induce *Id1* gene expression in *Bmpr2* ^*Δtd/+*^ PaSMCs, as well as the ability of BMP4 to induce *Smad6* gene expression in *Bmpr2* ^*Δtd/+*^ and *Bmpr2* ^*Δtd/del*^ PaSMCs (Figure 4). These findings show that Acvr2a is not required for BMP4 signaling in *Bmpr2* ^*Δtd/+*^ or *Bmpr2* ^*Δtd/del*^ PaSMCs. In contrast, silencing *Acvr2a* mRNA modestly decreased the ability of BMP7 to induce *Id1* and *Smad6* gene expressions in *Bmpr2* ^*Δtd/del*^ PaSMCs (Figure 4), as well as decreasing the ability of BMP7 to induce *Id1* gene expression in *Bmpr2* ^*Δtd/+*^ PaSMCs. These findings show that Bmpr2‑ΔTD and, to a lesser extent, Acvr2a can transduce BMP7 signaling in PaSMCs harboring the *Bmpr2* ^*Δtd*^ allele.

### Alk2 is required for the response of Bmpr2^Δtd/+^ PaSMCs to BMP7

**Figure 5:**
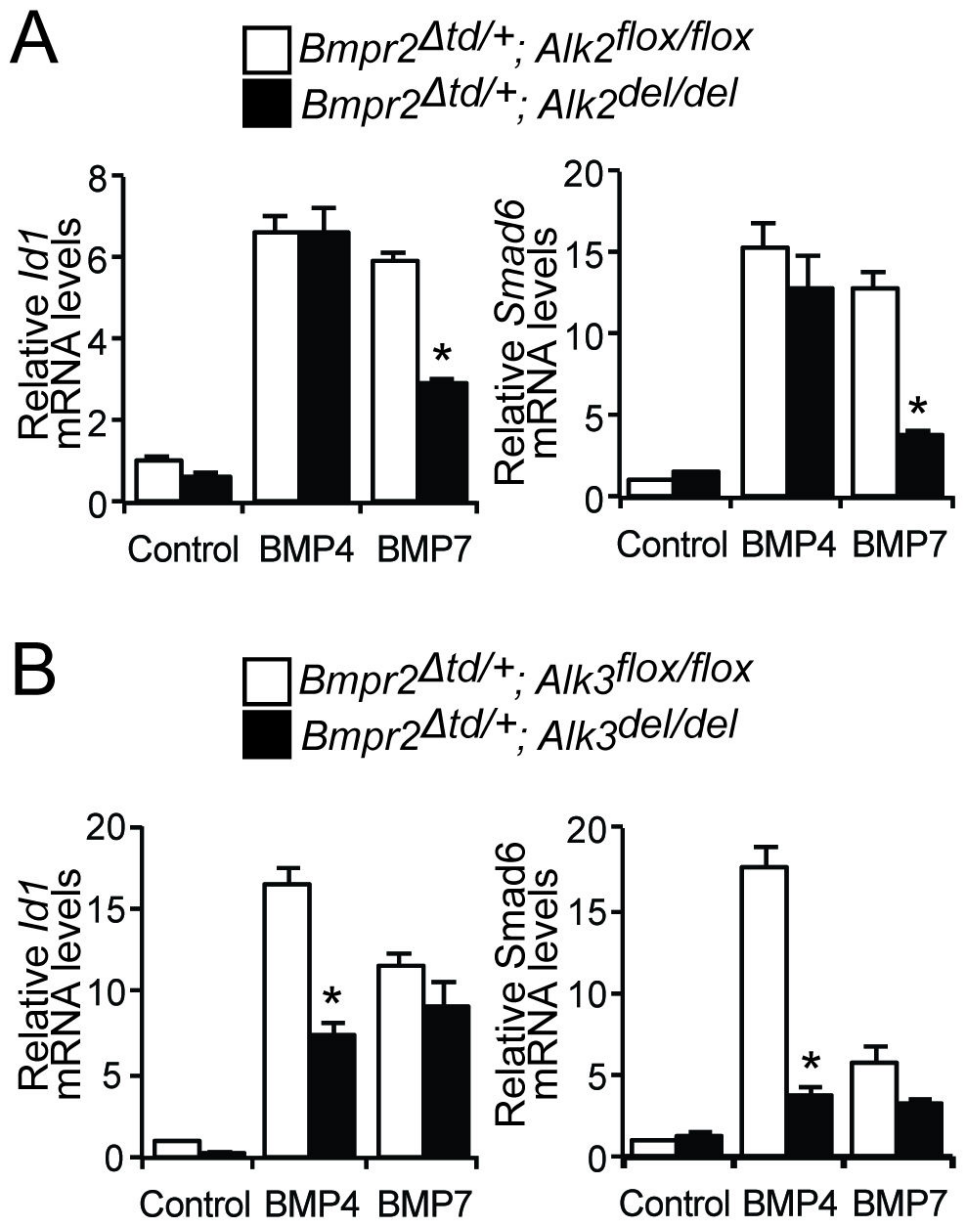
BMP7 preferentially utilizes Alk2 in *Bmpr2* ^*Δtd/+*^ PaSMCs. (A) The ability of BMP4 or BMP7 (10 ng/ml for 1.5 h) to induce *Id1* and *Smad6* gene expression, in *Bmpr2* ^*Δtd/+*^ PaSMCs deficient in Alk2 or expressing Alk2 was examined by qPCR. *Id1* and *Smad6* gene expression was normalized to *Gapdh* and expressed as fold-change relative to *Bmpr2* ^*Δtd/+*^ *; Alk2* ^*flox/flox*^ PaSMCs. *P < 0.01 compared to *Bmpr2* ^*Δtd/+*^ *; Alk2* ^*flox/flox*^ PaSMCs treated with BMP7. (B) The ability of BMP4 or BMP7 (10 ng/ml for 1.5 h) to induce *Id1* and *Smad6* gene expression, in *Bmpr2* ^*Δtd/+*^ PaSMCs deficient in Alk3 or expressing Alk3 was measured by qPCR. *Id1* and *Smad6* gene expression was normalized to *Gapdh* and expressed as fold-change relative to *Bmpr2* ^*Δtd/+*^ *; Alk3* ^*flox/flox*^ PaSMCs. *P < 0.01 compared to *Bmpr2* ^*Δtd/+*^ *; Alk3* ^*flox/flox*^ PaSMC treated with BMP4.

**Figure 6:**
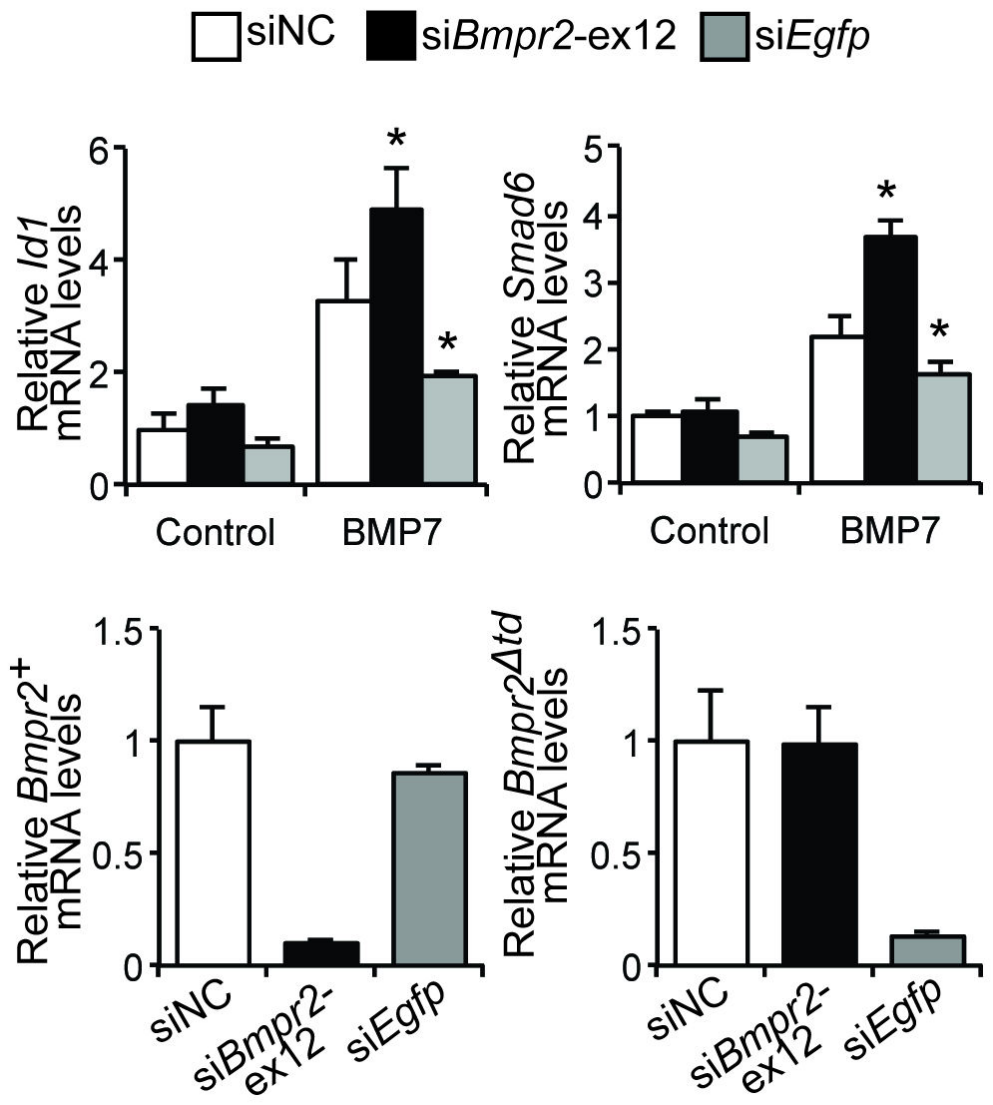
Bmpr2‑TD attenuates Alk2‑mediated BMP7 signaling in PaSMCs. Alk3‑deficient *Bmpr2* ^*Δtd/+*^ PaSMCs were transfected with specific siRNA to silence *Bmpr2* ^*+*^ (si*Bmpr2*‑ex12) or *Bmpr2* ^*Δtd*^ (si*Egfp*) transcripts. After 48 h, the ability of BMP7 to induce *Id1* and *Smad6* gene expression was measured by qPCR, normalized to *Gapdh* and expressed as fold-change relative to *Bmpr2* ^*Δtd/+*^; *Alk3* ^*del/del*^ PaSMCs treated with siNC. *P < 0.01 compared to control cells (siNC) treated with BMP7. Silencing efficiency was quantified by qPCR.

Based on our previous findings in *Bmpr2* ^*del/del*^ PaSMCs [5] and the high affinity of BMP7 for Alk2 [19,20], we hypothesized that the response to BMP7 would be less in *Bmpr2* ^*Δtd/+*^ cells deficient in Alk2 than in *Bmpr2* ^*Δtd/+*^ cells that express Alk2. To test this hypothesis, we generated *Bmpr2* ^*Δtd/+*^ mice carrying *Alk2*, *Alk3*, or both alleles flanked by loxP sequences. PaSMCs from these mice were infected with Ad-Cre to delete alleles flanked by loxP sequences or Ad-RFP as a control. Incubation with BMP4 induced *Id1* and *Smad6* gene expression similarly in *Bmpr2* ^*Δtd/+*^ *; Alk2* ^*flox/flox*^ and *Bmpr2* ^*Δtd/+*^; *Alk2* ^*del/del*^ PaSMCs (Figure 5A). In contrast, incubation with BMP4 induced *Id1* and *Smad6* gene expression less in *Bmpr2* ^*Δtd/+*^ *; Alk3* ^*del/del*^ than in *Bmpr2* ^*Δtd/+*^ *; Alk3* ^*flox/flox*^ PaSMCs (Figure 5B). The ability of BMP7 to induce *Id1* and *Smad6* gene expression was markedly less in *Bmpr2* ^*Δtd/+*^ *; Alk2* ^*del/del*^ than in *Bmpr2* ^*Δtd/+*^ *; Alk2* ^*flox/flox*^ PaSMCs and was similar in *Bmpr2* ^*Δtd/+*^ *; Alk3* ^*flox/flox*^ and *Bmpr2* ^*Δtd/+*^ *; Alk3* ^*del/del*^ PaSMCs (Figure 5). These results suggest that the enhanced responsiveness of *Bmpr2* ^*Δtd/+*^ PaSMCs to BMP7 requires the presence of Alk2. To corroborate these results, we examined the contribution of Bmpr2‑ΔTD or Bmpr2‑WT to mediate BMP7 signaling in PaSMCs predominantly expressing Alk2 (i.e. in Alk3‑deficient PaSMCs). Silencing of *Bmpr2* ^*+*^ transcripts in *Bmpr2* ^*Δtd/+*^ *; Alk3* ^*del/del*^ PaSMCs augmented the ability of BMP7 to induce *Id1* or *Smad6* gene expression, whereas silencing of *Bmpr2* ^*Δtd*^ transcripts reduced the responsiveness of *Bmpr2* ^*Δtd/+*^ *; Alk3* ^*del/del*^ PaSMCs to BMP7 (Figure 6). These results support the concept that BMP7 signaling is transduced by the mutant receptor Bmpr2‑ΔTD and Alk2, and suggest that the tail domain of Bmpr2 may inhibit BMP7 signaling via Alk2. Deletion of both Alk2 and Alk3 abrogated BMP4 and BMP7 signaling in *Bmpr2* ^*Δtd/+*^ PaSMCs, as well as in *Bmpr2* ^*+/+*^ PaSMCs (Figure S5). These findings suggested that the very low levels of Alk6 detected in PaSMCs are insufficient to transduce BMP signaling.

## Discussion

In this study, we report the generation of a genetically modified mouse that carries a *Bmpr2* allele with an NMD-resistant mutation in the sequences encoding the Bmpr2‑TD. We found that *Bmpr2* ^*Δtd/Δtd*^ mice die early in embryogenesis (E7.5‑8.5) and that *Bmpr2* ^*Δtd/+*^ mice appear to grow normally. RVSP is similar in *Bmpr2* ^*Δtd/+*^ mice and their WT littermates at 6 to 8 months of age. We observed that the receptor encoded by the mutant allele, Bmpr2‑ΔTD, is expressed and traffics to the membrane of PaSMCs. In PaSMCs from *Bmpr2* ^*Δtd/+*^ mice, we found a BMP7‑specific gain of signaling with preserved BMP4 signaling. Knockdown of *Bmpr2* ^*+*^ transcripts in *Bmpr2* ^*Δtd/+*^ PaSMCs or deletion of the *Bmpr2* ^*flox*^ allele in conditional *Bmpr2* ^*Δtdlflox*^ PaSMCs showed that Bmpr2‑WT is not required for these cells to transduce signaling in response to BMP7. However, knockdown of *Bmpr2* ^*Δtd*^ transcripts in *Bmpr2* ^*Δtd/+*^ PaSMCs inhibited BMP7 signaling. Finally, we determined that the increased responsiveness of *Bmpr2* ^*Δtd/+*^ PaSMCs to BMP7 relies on the presence of Alk2, thus revealing that the tail domain of Bmpr2 inhibits Alk2‑mediated signaling.

In the process of posttranscriptional regulation, the mRNA surveillance mechanism of NMD plays a critical role degrading aberrant transcripts prior to translation [16,17]. It was conceivable that transcripts generated by the *Bmpr2* ^*Δtd*^ allele would undergo NMD. We observed, however, that *Bmpr2* ^*Δtd*^ mRNA represented half of the *Bmpr2* transcripts expressed in *Bmpr2* ^*Δtd/+*^ PaSMCs. Likewise, Bmpr2‑ΔTD protein expression was readily detected in lysates from PaSMCs expressing the *Bmpr2* ^*Δtd*^ allele. These data show that transcripts from the *Bmpr2* ^*Δtd*^ allele were resistant to NMD. Our mouse model differs from other genetically modified mice carrying mutations in the Bmpr2-TD. Mutant transcripts from heterozygous mice carrying a *BMPR2* R899X knockin allele were found to be subject to NMD [21], rendering these knockin mice similar to haploinsufficient *Bmpr2* ^*+/-*^ mice. In contrast, BMPR2 R899X protein was detected in the pulmonary vasculature of mice in which a transgene specifying the mutant protein was inducibly overexpressed in smooth muscle cells [22].

Mice with *Bmpr2* mutations have been used to study how human *BMPR2* mutations might predispose carriers to PAH. *Bmpr2* ^*+/-*^ mice express about 50% of *Bmpr2* ^*+*^ mRNA levels and manifest little [18] or no [23,24] pulmonary hypertension at baseline; however, pulmonary hypertension induced by an inflammatory stress [24] or an infusion of serotonin [23] is more marked in *Bmpr2* ^*+/-*^ than in WT mice. Mice carrying one copy of a mutant *Bmpr2* allele lacking exon 2 (*Bmpr2* ^*ΔE2*^) do not manifest pulmonary hypertension at baseline but develop more marked pulmonary hypertension after prolonged exposure to hypoxia [25]. Although the main objective of our work was to study the role of the tail domain of Bmpr2 using cells from *Bmpr2* ^*Δtd/+*^ mice, we did examine whether *Bmpr2* ^*Δtd/+*^ mice spontaneously develop pulmonary hypertension. At baseline, RVSP does not differ in 6- to 8-month-old *Bmpr2* ^*Δtd/+*^ and WT mice. The absence of pulmonary hypertension at baseline in mice carrying heterozygous *Bmpr2* mutations (with mutant *Bmpr2* alleles expressed at levels similar to those of the WT allele) is consistent with the observation that PAH occurs in only one-fifth of the individuals harboring *BMPR2* mutations, suggesting that additional genetic or environmental factors (second hit) are involved in the clinical manifestation of the disease [14].

BMPs are involved in numerous processes during early embryonic development including organogenesis and morphogenesis [1]. We previously demonstrated that *Bmpr2* ^*-/-*^ embryos are arrested during gastrulation [26]. In contrast, mice homozygous for a hypomorphic Bmpr2 (*Bmpr2* ^*ΔE2/ΔE2*^), which appears to retain some BMP signaling capabilities, are able to complete gastrulation, but die during midgestation due to defects in the organogenesis of the cardiovascular and skeletal systems [27]. In the present study, we observed that homozygous *Bmpr2* ^*Δtd/Δtd*^ mice die in gastrulation even though the Bmpr2‑ΔTD mutant retains the ability to activate Smads in response to BMP ligands. These observations show that embryogenesis not only requires Bmpr2 kinase activity, but also the presence of the tail domain of Bmpr2.

To begin to understand how BMP signaling is modulated by the absence of the tail domain of Bmpr2, we tested the responsiveness of *Bmpr2* ^*Δtd/+*^ PaSMCs to BMP4 and BMP7 and found an unexpected BMP7‑specific gain of function. We considered several possible mechanisms by which this gain of function might occur. First, we considered the possibility that the absence of the Bmpr2‑TD would alter the ability of the receptor to traffic and localize to the cell membrane. In *Xenopus* embryos, the neuroectodermal protein Jiraiya interacts with a motif in the Bmpr2‑TD to inhibit bmpr2 trafficking to the cell membrane [28], suggesting that loss of the tail domain may facilitate the trafficking of the receptor to the cellular membrane. Moreover, it has been reported that BMPR2 proteins with mutations in the tail domain can traffic to the cellular membrane and can transduce BMP signaling [29,30]. Similarly, we observed that Bmpr2‑ΔTD localized to the cell membrane of PaSMCs. Taken together, these findings demonstrate that the Bmpr2‑TD is not required for trafficking of the receptor to the cell surface.

Previous reports have identified several proteins that can interact with the tail domain of BMPR2 and regulate BMP signaling. Tribbles homolog 3 (Trib3) interacts with the BMPR2-TD and dissociates from the receptor upon BMP4 binding and activation of the receptor complex [8]. Once unbound, Trib3 promotes the ubiquitination of SMURF1 (SMAD-specific E3 ubiquitin-protein ligase 1), thereby enhancing BMP signaling by reducing the degradation of activated SMADs. Another protein interacting with the BMPR2-TD, cGMP-dependent protein kinase type I (PKG), phosphorylates the receptor leading to enhanced BMP signaling [9]. Following BMP2 binding to the receptor complex, PKG dissociates from the BMPR2-TD and binds to activated SMADs and enhances their transcriptional activity. However, loss of Trib3 or PKG binding to the tail domain of BMPR2 is unlikely to explain the BMP7‑specific increased responsiveness of cells expressing Bmpr2‑ΔTD.

We previously reported that Acvr2a transduced BMP4 signaling and was required for the enhanced BMP7 signaling found in PaSMCs lacking Bmpr2 (*Bmpr2* ^*del/del*^) [5]. We considered the possibility that the BMP7‑specific gain of function seen in PaSMCs carrying the *Bmpr2* ^*Δtd*^ allele was exclusively transduced by Acvr2a. We observed, however, that silencing of the *Bmpr2* ^*Δtd*^ allele in *Bmpr2* ^*Δtd/+*^ PaSMCs markedly reduced BMP7 signaling. Moreover, silencing *Acvr2a* transcripts only modestly affected the ability of *Bmpr2* ^*Δtd/+*^ or *Bmpr2* ^*Δtd/del*^ PaSMCs to transduce BMP7 signaling. These results demonstrate that the enhanced BMP7 signaling seen in PaSMCs carrying the *Bmpr2* ^*Δtd*^ allele is predominantly mediated by Bmpr2‑ΔTD rather than by Acvr2a. We previously reported that knockdown of Acvr2a expression reduced BMP signaling in *Bmpr2* ^*del/del*^ PaSMC but not in *Bmpr2* ^*flox/flox*^ cells. In our current studies, we observed that BMP7 signaling was greater in *Bmpr2* ^*Δtd/del*^ PaSMCs than in *Bmpr2* ^*Δtd/flox*^ PaSMCs. Taken together, these observations suggest that the tail domain of Bmpr2 can suppress BMP7 signaling transduced by either Acvr2a or Bmpr2‑ΔTD.

Different BMPs have distinct affinities for each of the BMP receptors. For example, BMP7 has a higher affinity for Alk2 than for other BMP type 1 receptors [19,20]. We therefore tested the hypothesis that the enhanced BMP7 signaling seen in cells expressing Bmpr2‑ΔTD is mediated by Alk2. In *Bmpr2* ^*Δtd/+*^ PaSMCs, deletion of *Alk3* markedly reduced BMP4 signaling but not BMP7 signaling. In contrast, we observed that deletion of *Alk2* markedly impaired the ability of Bmpr2‑ΔTD to transduce BMP7 signals. These results demonstrate that Bmpr2‑ΔTD and Alk2 mediate BMP7 signaling in cells harboring the *Bmpr2* ^*Δtd*^ allele. Taken together with our observations in *Bmpr2* ^*del/del*^ PaSMC, these findings suggest that the tail domain of Bmpr2 suppresses Alk2‑dependent BMP7 signaling by either Bmpr2‑ΔTD or Acvr2a and raise the possibility that the the tail domain of Bmpr2 directly inhibits Alk2 function. Unfortunately, currently available commercial antibodies detect BMP type I receptors only when they are overexpressed, hampering the detection of interactions of endogenously expressed BMP receptors.

In conclusion, we report the generation of a mouse harboring an NMD-resistant mutation in the sequences encoding for the Bmpr2‑TD. Mice homozygous for the mutant allele died early in embryogenesis, possibly because of a critical role for Bmpr2‑TD in gastrulation. Heterozygous mice grow normally and, as observed in genetically modified mice carrying other mutant Bmpr2 alleles, they did not spontaneously develop PAH. The BMP7‑specific gain of function observed in PaSMCs from heterozygous *Bmpr2* ^*Δtd/+*^ mice was mediated by the mutant receptor and the BMP type 1 receptor, Alk2. Our data suggest that BMP7 signaling is inhibited in WT PaSMCs by a restriction exerted by the Bmpr2‑TD over Alk2. These data also raise the possibility that some disease-causing *BMPR2* mutations may alter BMP signaling in a BMP ligand-specific manner. 

## Material and Methods

### Generation of mice carrying mutant BMP receptors

The strategy to create the *Bmpr2* ^*Δtd*^ allele is shown in Figure S1A. The *Bmpr2* ^*Δtd*^-targeting vector carries sequences for *Egfp* (in frame after exon 11), the SV40 polyadenylation signal (SV40pA), and a phosphoglycerol kinase promoter-controlled neomycin-resistance gene (PGK-neo) cassette after exon 11. Mouse embryonic stem (ES) cells were transfected with the targeting vector, and Southern blot analysis identified an ES cell clone with homologous recombination (Figure S1B). The recombinant ES cells were injected into blastocysts and germline transmission was achieved. Removal of the PGK-neo cassette, flanked by loxP sequences, was achieved by mating the heterozygous *Bmpr2* ^*Δtd/+*^ mice with Ella-Cre transgenic mice. Mice heterozygous for the *Bmpr2* ^*Δtd*^ allele were derived from crossing chimeric mice with C57BL/6 female mice. PCR analysis for genotyping purposes using DNA isolated from E7.5 embryos is shown in Figure S1C. Genotyping primers for the mutant *Bmpr2* ^*Δtd*^ allele are 5’-GTGCTACAGGCAGTGAGGTCACTC-3’ and 5’-TAGGTCAGGGTGGTCACGAGGGTG-3’ (400-bp product). Genotyping primers for *Bmpr2* ^*+*^ allele are 5’-GACTTCACACAGGCTGCAAATGGG-3’ and 5’-CATACTGGGTTGTGGCAGCATGGG-3’ (300-bp product).

*Bmpr2* ^*Δtd/+*^ mice were backcrossed more than 9 times onto a C57BL/6 background. *Bmpr2* ^*flox/flox*^ mice [31] bred onto C57BL/6 background were bred to *Bmpr2* ^*Δtd/+*^ mice to generate *Bmpr2* ^*Δtd/flox*^ mice. *Alk2* ^*flox/flox*^ mice on a mixed C57BL/6; SV129 background [32] or *Alk3* ^*flox/flox*^ mice on a C57BL/6 background [33] were bred to *Bmpr2* ^*Δtd/+*^ mice to generate *Bmpr2* ^*Δtd/+*^ *; Alk2* ^*flox/flox*^, or *Bmpr2* ^*Δtd/+*^ *; Alk3* ^*flox/flox*^ mice, respectively.

All animal experiments were conducted under protocols reviewed and approved by the Subcommittee on Research and Animal Care of the Massachusetts General Hospital.

### Hemodynamic measurements in Bmpr2^Δtd/+^ and WT mice

Mice were anesthetized with ketamine (100 mg/kg) and fentanyl (250 µg/kg) intraperitoneally, intubated, and mechanically ventilated (10 µl/g, 100 breaths per minute; FiO_2_ = 1). Pancuronium (2 mg/kg) was administered intraperitoneally, and a PE‑10 polyethylene catheter was placed in the left carotid artery for continuous measurement of heart rate and systemic arterial pressure. A 1.2F high-fidelity pressure catheter (FTS‑1211B‑0018; Scisense, London, ON, Canada) was advanced into the right ventricle via the jugular vein to measure right ventricular systolic pressure (RVSP), as an estimate of pulmonary arterial systolic pressure. All signals were recorded and analyzed using a data acquisition system (A D Instruments, Colorado Springs, CO). At the end of the study mice were euthanized with an intraperitoneal injection of pentobarbital (200 mg/kg).

### PaSMC isolation and culture

Mice were euthanized with an intraperitoneal injection of pentobarbital (200 mg/kg). Pulmonary arteries (PA) were isolated and incubated individually in trypsin‑EDTA for 10 min at 37°C. PAs were cut into ~1 mm^3^ pieces and enzymatically digested using a solution of collagenase, papain, elastase, and soybean trypsin inhibitor for 30 min at 37°C. After dissociation, cells were washed twice in DMEM containing 20% fetal bovine serum. After the final wash, cells were resuspended in DMEM with 20% FBS and antibiotics (penicillin – streptomycin) and were cultured at 37°C in 10% CO_2_. After the first passage, cells were grown in DMEM containing 10% FBS. Cells were used for experiments between passages 3 and 10.

### Adenovirus infection

To disrupt *Bmpr2*, *Alk2*, or *Alk3* genes in PaSMCs isolated from mice harboring alleles carrying loxP sequences, cells were infected with Ad-Cre or Ad-RFP, as a control, at a multiplicity of infection of 150. After cells recovered from infection, efficiency of recombination of the *Bmpr2* allele flanked by loxP sequences was determined by PCR and immunoblot techniques, as reported previously [31]. Efficiency of recombination of *Alk2* or *Alk3* alleles flanked by loxP sequences was determined by qPCR using hydrolysis probes.

### Small interfering RNA inhibition of BMP receptors

Silencer® Select siRNA (Applied Biosystems, Life Technologies) specific for *Bmpr2*, *Egfp*, and *Acvr2a* or negative control siRNA (30-50 nM) were transfected into PaSMCs using Pepmute siRNA transfection reagent (SignaGen Laboratories), as described by the manufacturer. After 48 hours, transfected cells were starved in DMEM with 0.1% FBS for 12 to 16 hours and then treated with BMP4 or BMP7.

### Gene expression

Total RNA was extracted by guanidine isothiocyanate/phenol method. cDNA was synthesized using M-MLV reverse transcriptase and random primers (Promega). *Id1*, *Smad6*, *Bmpr2*, *Egfp*, *Acvr2a*, *Alk2*, *Alk3* and *Gapdh* transcript levels were measured by qPCR in a Mastercycler ep realplex 2 (Eppendorf) using hydrolysis probes (TaqMan^®^ Gene Expression Assays, Applied Biosystems, Life Technologies) and Probe Fast Master Mix (Kapa Biosystems). qPCR reactions were prepared using the specific FAM-labeled hydrolysis probes and the *Gapdh* VIC-labeled primer-limited hydrolysis probe, as internal reference gene. Changes in relative gene expression normalized to *Gapdh* mRNA levels were determined using the relative C_T_ method.

### Immunoblot techniques

Confluent PaSMCs were incubated with DMEM with 0.1% FBS for 12 to 16 hours and then treated for various times with BMP4 or BMP7. Cells were lysed with RIPA buffer containing proteinase and phosphatase inhibitor cocktails (Sigma). Lysates were mixed with NuPAGE^®^ LDS sample buffer (Invitrogen, Life Technologies) containing 1mM DTT. Proteins were separated by NuPAGE® Bis‑Tris gels (Invitrogen, Life Technologies), transferred to polyvinylidene difluoride membranes (Immobilon‑FL, Millipore), and blocked in TBS containing 5% skim milk and 0.1% Tween 20. Membranes were reacted with antibodies directed against phosphorylated Smad1/5/8, Gapdh (Cell Signaling); as well as total Smad1/5/8 (Santa Cruz Biotechnology), the tail domain of Bmpr2 (BD Transduction Laboratories), or GFP (Roche). After incubation with HRP-conjugated IgG secondary antibodies (Epitomics and Cell Signaling) and ECL Plus reagent (GE Healthcare Life Sciences), chemifluorescence signals were detected with a Versadoc® Imaging Systems. Captured images were analyzed with ImageJ software (NIH).

### Immunofluorescence labeling

PaSMCs were transiently transfected with a plasmid carrying the sequences of Bmpr2‑ΔTD. After 16 hours, cells were fixed with 4% paraformaldehyde in PBS and permeabilized with 0.1% Triton X-100 in PBS. Immunohistochemical staining was performed with a mouse anti-GFP antibody (Invitrogen, Life Technologies), followed by incubation with fluorescein isothiocyanate-labeled goat anti-mouse IgG. Subcellular localization of Bmpr2‑ΔTD was determined by confocal laser scanning microscopy.

### Statistics

Differences between groups were determined using two-way ANOVA for experiments using cultured PaSMCs. Figures show results representative of three or more PaSMC isolates for each genotype. For qPCR experiments, each experimental condition was performed in quadruplicate. All data are expressed as means ± standard deviation. The Student t‑test was used to analyze mouse hemodynamic measurements (HR, MAP, RVSP). A value of p < 0.05 indicated a significant difference. 

## Supporting Information

Figure S1 ***Bmpr2*^*Δtd*^ gene-targeting strategy.** (A) Schematic diagrams (from top to bottom) of the wild-type *Bmpr2* gene, the targeting vector, and the mutant *Bmpr2* ^*Δtd*^ allele after homologous recombination. The entire tail domain of Bmpr2 is encoded by exon 12 and 13. A genomic fragment containing intron 11 and exon 12 was replaced by the sequence of Egfp (in frame after exon 11) followed by SV40 polyA signal and a PGK-neo cassette. (B) Southern blot analysis of DNA isolated from ES clones. (C) PCR genotyping analysis of E7.5 embryos generated by intercrosses of F1 heterozygotes.
(TIF)

Figure S2 **Hemodynamic measurements in *Bmpr2*^*+/+*^ and *Bmpr2*^*Δtd/+*^ mice.** Heart rate (HR), mean systemic arterial pressure (MAP), and right ventricular systolic pressure (RVSP) were measured in 6- to 8-month-old mice (littermates).
(TIF)

Figure S3 *Id1* gene expression in *Bmpr2*^*+/+*^ and *Bmpr2*^*Δtd/+*^ PaSMCs after 24 hours treatment with BMP4 or BMP7 (10 ng/ml; *p < 0.01 versus without BMP ligand).
(TIF)

Figure S4 **Immunoblotting of *Bmpr2*^*Δtd/flox*^ and *Bmpr2*^*Δtd/del*^ PaSMCs with anti-Bmpr2‑TD to detect Bmpr2‑WT or anti-GFP to detect Bmpr2‑ΔTD.** Gapdh was used as loading control.
(TIF)

Figure S5 **Concomitant loss of Alk2 and Alk3 prevents BMP signaling in PaSMCs.** (A) *Bmpr2* ^*∆td/+*^ *; Alk2* ^*flox/flox*^ *; Alk3* ^*flox/flox*^ [+] or *Bmpr2* ^*∆td/+*^ *; Alk2* ^*del/del*^ *; Alk3* ^*del/del*^ [-] PaSMCs were stimulated with BMP4 or BMP7 (10 ng/ml) for 30 min or 1 h. Immunoblotting for pSmad1/5/8 and Smad1/5/8 show that *Bmpr2* ^*∆td/+*^ PaSMCs lacking the expression of Alk2 and Alk3 have lost the ability to phosphorylate BMP-responsive Smad1/5/8. (B) *Bmpr2* ^*+/+*^ *; Alk2* ^*flox/flox*^ *; Alk3* ^*flox/flox*^ or *Bmpr2* ^*+/+*^ *; Alk2* ^*del/del*^ *; Alk3* ^*del/del*^ PaSMCs were stimulated with BMP4 or BMP7 (10 ng/ml) for 2 h, and the ability to induce *Id1* and *Smad6* gene expression was measured by qPCR. *Bmpr2* ^*+/+*^ PaSMCs lacking expression of Alk2 and Alk3 have lost the ability to induce *Id1* and *Smad6* gene expression in response to BMP ligands. (C) *Bmpr2* ^*∆td/+*^ *; Alk2* ^*flox/flox*^ *; Alk3* ^*flox/flox*^ or *Bmpr2* ^*∆td/+*^ *; Alk2* ^*del/del*^ *; Alk3* ^*del/del*^ PaSMCs were stimulated with BMP4 or BMP7 (10 ng/ml) for 2 h, and the ability to induce *Id1* and *Smad6* gene expression was measured by qPCR. *Bmpr2* ^*∆td/+*^ PaSMCs lacking expression of Alk2 and Alk3 have lost the ability to induce *Id1* and *Smad6* gene expression in response to BMP ligands.
(TIF)
