# Supplementary figures and images for: Deletion of the Sequence Encoding the Tail Domain of the Bone Morphogenetic Protein type 2 Receptor Reveals a Bone Morphogenetic Protein 7-Specific Gain of Function
Source: PLoS One. 2013 Oct 8;8(10):e76947. doi: 10.1371/journal.pone.0076947 (PMC3792867; doi:10.1371/journal.pone.0076947)

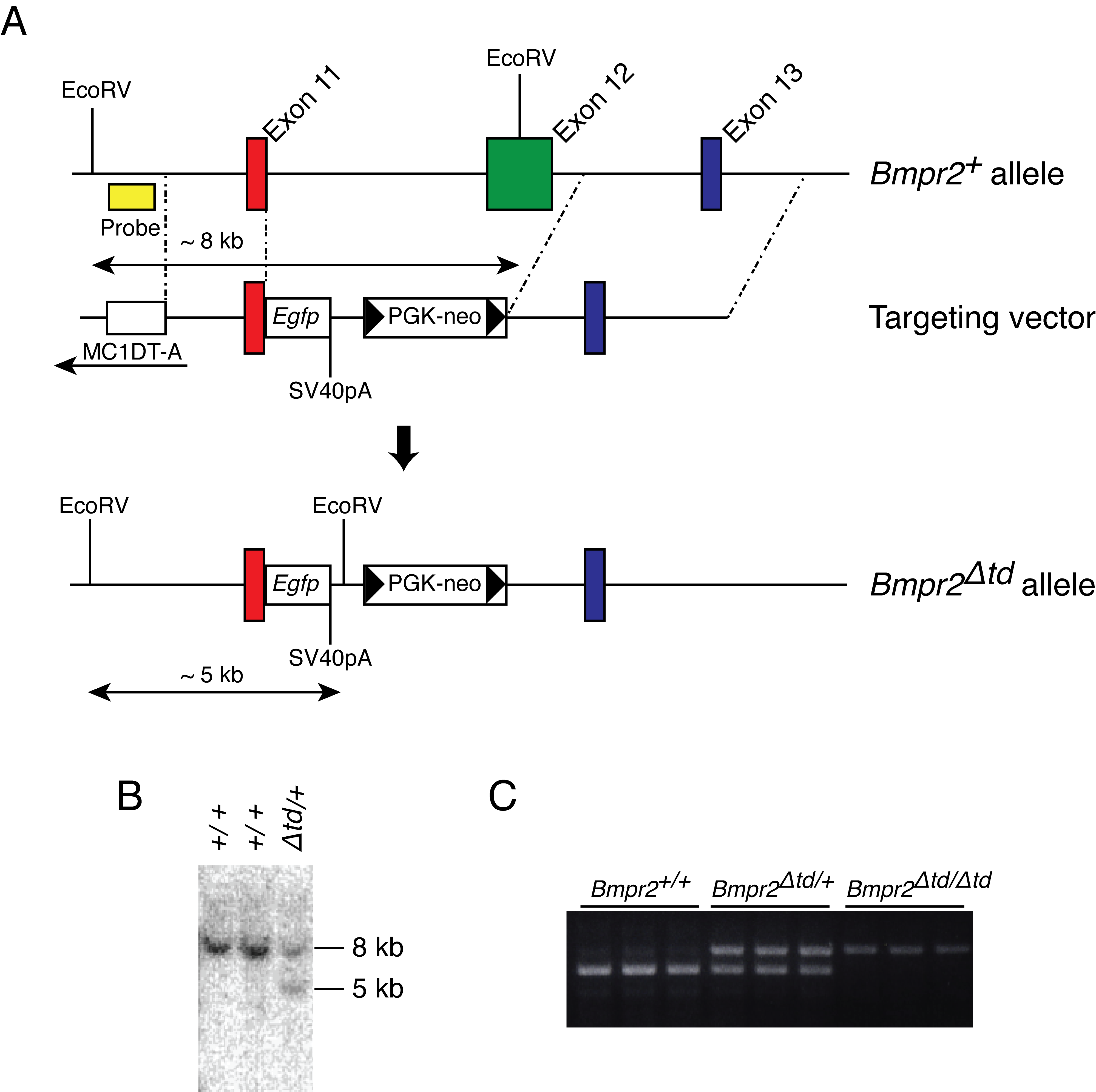

Supplement: Figure S1 — Bmpr2Δtd gene-targeting strategy. (A) Schematic diagrams (from top to bottom) of the wild-type Bmpr2 gene, the targeting vector, and the mutant Bmpr2 Δtd allele after homologous recombination. The entire tail domain of Bmpr2 is encoded by exon 12 and 13. A genomic fragment containing intron 11 and exon 12 was replaced by the sequence of Egfp (in frame after exon 11) followed by SV40 polyA signal and a PGK-neo cassette. (B) Southern blot analysis of DNA isolated from ES clones. (C) PCR genotyping analysis of E7.5 embryos generated by intercrosses of F1 heterozygotes. (TIF) [file pone.0076947.s001.tif]

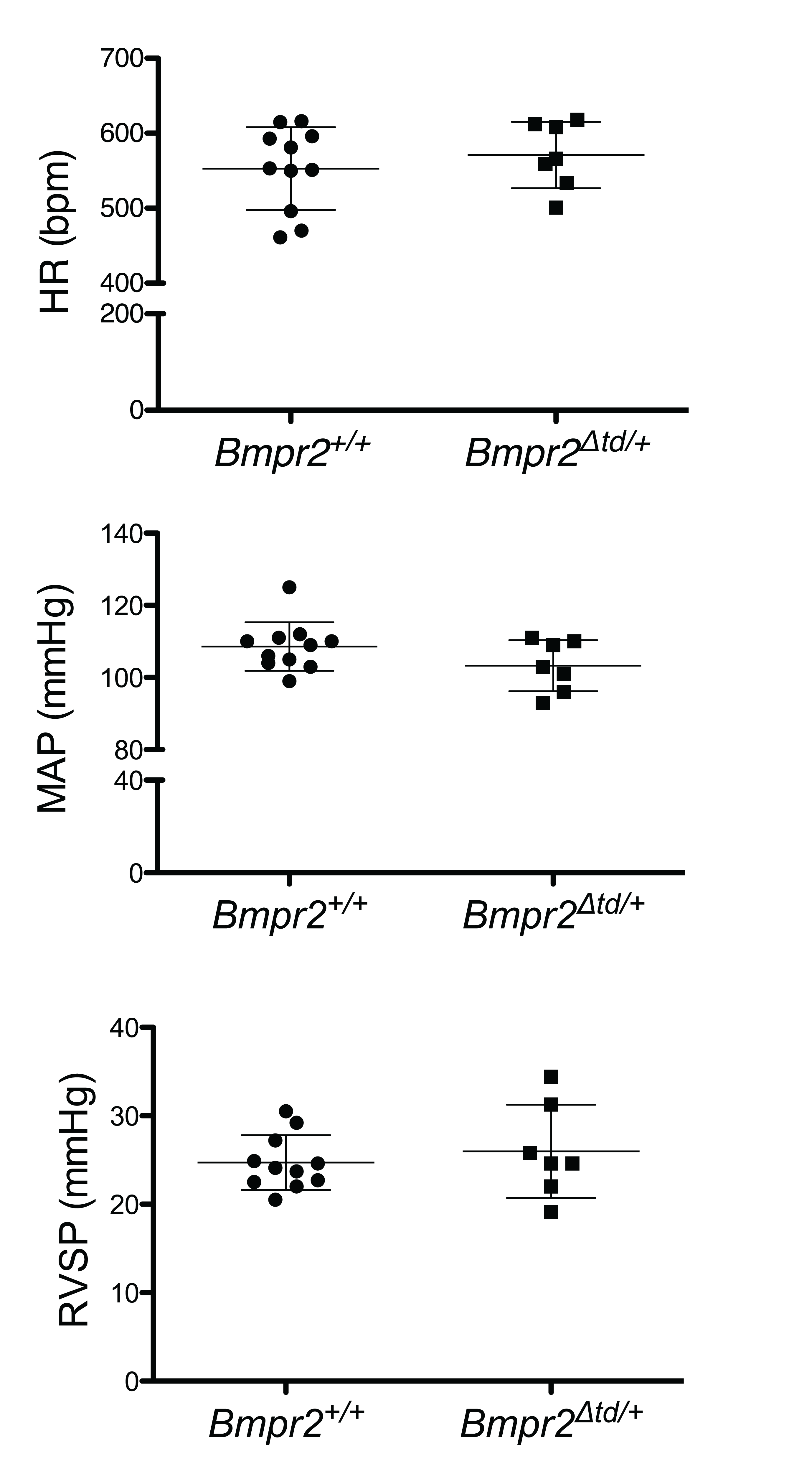

Supplement: Figure S2 — Hemodynamic measurements in Bmpr2+/+ and Bmpr2Δtd/+ mice. Heart rate (HR), mean systemic arterial pressure (MAP), and right ventricular systolic pressure (RVSP) were measured in 6- to 8-month-old mice (littermates). (TIF) [file pone.0076947.s002.tif]

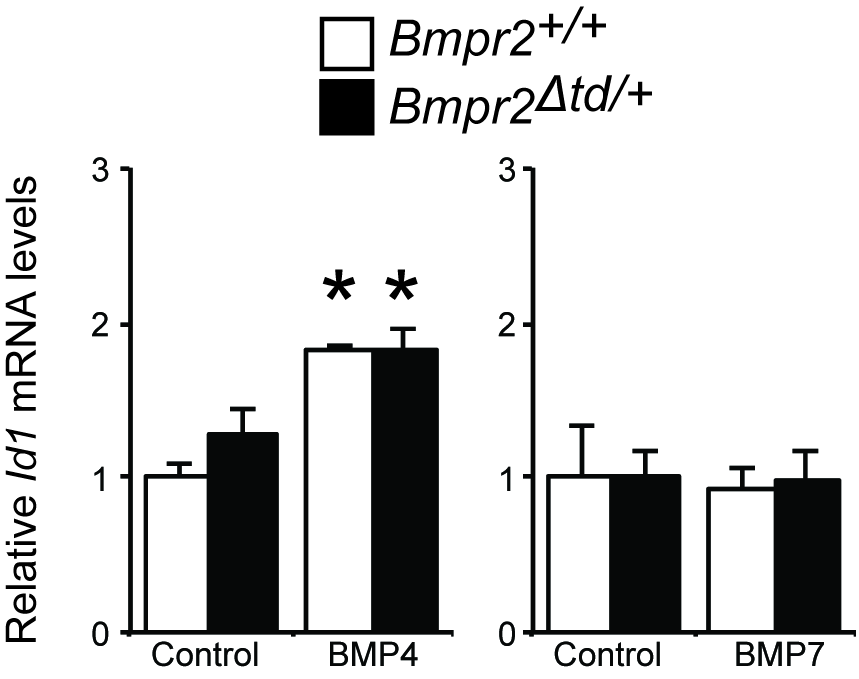

Supplement: Figure S3 — Id1 gene expression in Bmpr2+/+ and Bmpr2Δtd/+ PaSMCs after 24 hours treatment with BMP4 or BMP7 (10 ng/ml; *p < 0.01 versus without BMP ligand). (TIF) [file pone.0076947.s003.tif]

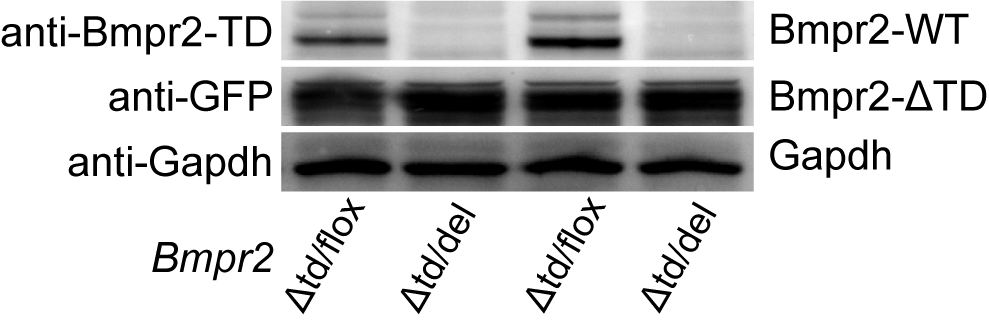

Supplement: Figure S4 — Immunoblotting of Bmpr2Δtd/flox and Bmpr2Δtd/del PaSMCs with anti-Bmpr2‑TD to detect Bmpr2‑WT or anti-GFP to detect Bmpr2‑ΔTD. Gapdh was used as loading control. (TIF) [file pone.0076947.s004.tif]

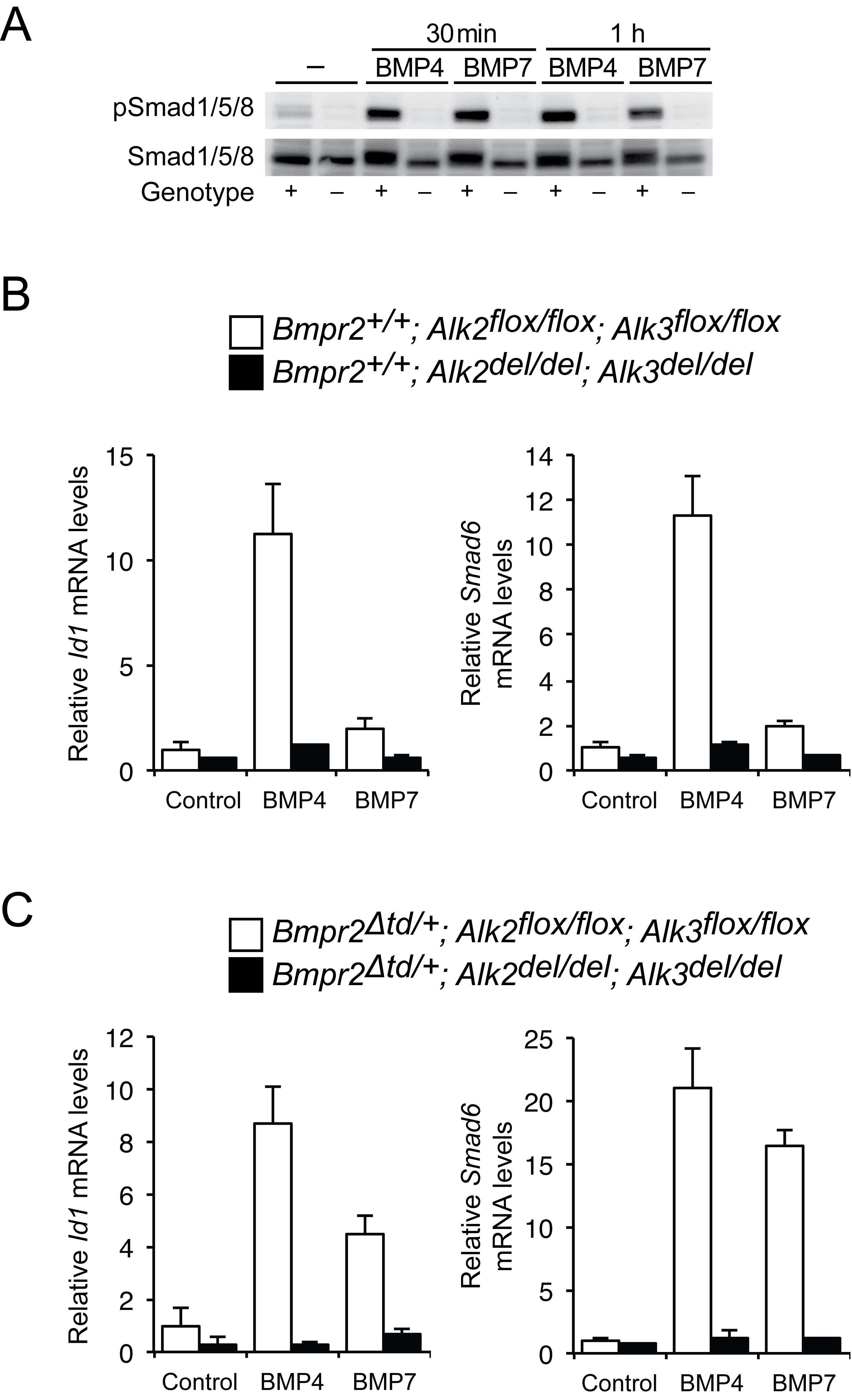

Supplement: Figure S5 — Concomitant loss of Alk2 and Alk3 prevents BMP signaling in PaSMCs. (A) Bmpr2 ∆td/+ ; Alk2 flox/flox ; Alk3 flox/flox [+] or Bmpr2 ∆td/+ ; Alk2 del/del ; Alk3 del/del [-] PaSMCs were stimulated with BMP4 or BMP7 (10 ng/ml) for 30 min or 1 h. Immunoblotting for pSmad1/5/8 and Smad1/5/8 show that Bmpr2 ∆td/+ PaSMCs lacking the expression of Alk2 and Alk3 have lost the ability to phosphorylate BMP-responsive Smad1/5/8. (B) Bmpr2 +/+ ; Alk2 flox/flox ; Alk3 flox/flox or Bmpr2 +/+ ; Alk2 del/del ; Alk3 del/del PaSMCs were stimulated with BMP4 or BMP7 (10 ng/ml) for 2 h, and the ability to induce Id1 and Smad6 gene expression was measured by qPCR. Bmpr2 +/+ PaSMCs lacking expression of Alk2 and Alk3 have lost the ability to induce Id1 and Smad6 gene expression in response to BMP ligands. (C) Bmpr2 ∆td/+ ; Alk2 flox/flox ; Alk3 flox/flox or Bmpr2 ∆td/+ ; Alk2 del/del ; Alk3 del/del PaSMCs were stimulated with BMP4 or BMP7 (10 ng/ml) for 2 h, and the ability to induce Id1 and Smad6 gene expression was measured by qPCR. Bmpr2 ∆td/+ PaSMCs lacking expression of Alk2 and Alk3 have lost the ability to induce Id1 and Smad6 gene expression in response to BMP ligands. (TIF) [file pone.0076947.s005.tif]
